# Conductive vial electromembrane extraction of opioids from oral fluid

**DOI:** 10.1007/s00216-023-04807-3

**Published:** 2023-06-29

**Authors:** Tonje Gottenberg Skaalvik, Chen Zhou, Elisabeth Leere Øiestad, Solfrid Hegstad, Roger Trones, Stig Pedersen-Bjergaard

**Affiliations:** 1 Department of Clinical Pharmacology, St. Olav University Hospital, Professor Brochs Gate 6, 7030 Trondheim, Norway; 2 Department of Pharmacy, University of Oslo, P.O. Box 1068 Blindern, 0316 Oslo, Norway; 3 West China School of Public Health and West China Fourth Hospital, Sichuan University, Chengdu, 610041 China; 4 Division of Laboratory Medicine, Department of Forensic Sciences, Oslo University Hospital, P.O. Box 4459 Nydalen, 0424 Oslo, Norway; 5 Extraction Technologies Norway, Verkstedveien 29, 1424 Ski, Norway; 6 Department of Pharmacy, Faculty of Health and Medical Sciences, University of Copenhagen, Universitetsparken 2, 2100 Copenhagen, Denmark

**Keywords:** Electromembrane extraction, Opioids, Oral fluid, Drug analysis, Liquid membrane

## Abstract

The use of oral fluid as sample matrix has gained significance in the analysis of drugs of abuse due to its non-invasive nature. In this study, the 13 opioids morphine, oxycodone, codeine, O-desmethyl tramadol, ethylmorphine, tramadol, pethidine, ketobemidone, buprenorphine, fentanyl, cyclopropylfentanyl, etonitazepyne, and methadone were extracted from oral fluid using electromembrane extraction based on conductive vials prior to analysis with ultra-high performance liquid chromatography–tandem mass spectrometry. Oral fluid was collected using Quantisal collection kits. By applying voltage, target analytes were extracted from oral fluid samples diluted with 0.1% formic acid, across a liquid membrane and into a 300 μL 0.1% (v/v) formic acid solution. The liquid membrane comprised 8 μL membrane solvent immobilized in the pores of a flat porous polypropylene membrane. The membrane solvent was a mixture of 6-methylcoumarin, thymol, and 2-nitrophenyloctyl ether. The composition of the membrane solvent was found to be the most important parameter to achieve simultaneous extraction of all target opioids, which had predicted log *P* values in the range from 0.7 to 5.0. The method was validated in accordance to the guidelines by the European Medical Agency with satisfactory results. Intra- and inter-day precision and bias were within guideline limits of ± 15% for 12 of 13 compounds. Extraction recoveries ranged from 39 to 104% (CV ≤ 23%). Internal standard normalized matrix effects were in the range from 88 to 103% (CV ≤ 5%). Quantitative results of authentic oral fluid samples were in accordance with a routine screening method, and external quality control samples for both hydrophilic and lipophilic compounds were within acceptable limits.

**Supplementary Information:**

The online version contains supplementary material available at 10.1007/s00216-023-04807-3.

## Introduction

Opioids are common analytes included in drug screening methodologies, as they are frequently misused or abused analgesics. The overarching group of drugs includes naturally occurring opiates, such as morphine and codeine, semi-synthetic opioids such as buprenorphine and synthetic opioids like methadone and fentanyl. The latest class of synthetic opioids to emerge on the illicit drug market are 2-Benzyl benzimidazole analogues, also known as *nitazenes* [1, 2].

Oral fluid (OF) has gained popularity as an alternative biological matrix for the detection of drugs in forensic and clinical settings, allowing simple and non-invasive sampling. The term OF refers to the mixture of saliva and other fluids and particles present in the oral cavity [3]. OF has been used for the analysis of a variety of drugs previously, including opioids [4–6]. Several reviews on the drug disposition, collection, interpretation and analysis of OF are available [7–9]. Sampling is often carried out using commercial collection devices that ensure adequate volume collection to a collector pad. The OF sampling kits contain surfactants, buffers, dyes and preservatives to improve analyte stability and elute analytes off the collector pad. Analysis of such samples thus usually includes an extraction step for sample clean-up prior to LC-MS analysis [7, 8].

Microextraction techniques have emerged as alternative sample preparation methods as part of the development towards smaller systems that consume less solvents and reagents. Electromembrane extraction (EME) is a form of membrane based liquid-phase microextraction (LPME) that incorporates an electric field to enhance mass transfer. Advantages are exhaustive extractions, sample clean-up and enrichment using only few microlitres (< 10 μL) of organic solvent, with faster kinetics compared to membrane based LPME [10, 11]. Since the introduction of EME in 2006 [12], extraction of organic and inorganic analytes from biological [13–16], environmental [17–19] and food samples [20–22] has been reported using a variety of technical formats. Operational modes and configurations are based on hollow fibres, 96-wells and microchip technology, among others, and have been summarized in multiple reviews [23–26]. A commercial device based on conductive vials was recently launched, which is considered a step closer towards routine implementation [27].

The typical EME system is a three-phase system consisting of an aqueous sample separated from an aqueous acceptor by a thin organic liquid membrane. Application of voltage (i.e. *extraction potential)* across the liquid membrane facilitates transfer of ionized compounds from the sample, across the liquid membrane and into the acceptor, which is collected for further analysis. Selectivity is tuned by altering the electric field (polarity and magnitude) and the physiochemical properties of the membrane. Highly selective extractions are thus possible with EME. Conditions favouring high selectivity are low-voltage [28] and liquid membranes with properties tuned towards analyte-specific interactions [29–31]. On the other end of the scale, low selectivity extractions that isolate a broad range of analytes from the sample matrix are relevant for applications such as multi-component drug screening. Finding robust EME conditions for extraction of compounds with a wide range of properties is not straightforward.

In theory, EME is applicable for all ionizable compounds; however, the application range is currently limited by suitable liquid membranes. In general, the membrane solvent should have minimal leakage to aqueous solution as this may lead to excessive current, which further negatively affects reproducibility due to electrolysis [32]. This is highly important, as robust systems are essential for routine clinical or forensic applications. In addition, the physiochemical properties of the liquid membrane must favour some degree of analyte partitioning for efficient extraction. Fundamental studies and reviews on the molecular interactions and application ranges of different liquid membranes can be found in the literature [33–36]. However, the selection of the liquid membrane remains to be by trial and error in most applications. To guide method development, recent work reported on generalized EME conditions aimed to characterize systematically the application range of membrane solvents using a large collection of model analytes [37].

The aim of the present study was to develop EME conditions for extraction of target opioids from OF and evaluate whether data quality was within recommended guidelines for bioanalytical methods. Target opioids were morphine, oxycodone, codeine, O-desmethyl tramadol (metabolite), ethylmorphine, tramadol, pethidine, ketobemidone, buprenorphine, fentanyl, cyclopropylfentanyl, etonitazepyne (N-pyrrolidino etonitazene) and methadone. The opioids all have basic functionality, which makes them applicable for simultaneous extraction with EME. However, lipophilicity varies, with predicted *n*-octanol-water partition coefficients (log *P*) ranging from 0.7 to 5.0 (Table S1), which complicates method development. EME was carried out using prototype equipment for a commercial EME device, employing conductive vials to house the sample and acceptor. The study aimed to find a suitable, robust liquid membrane for opioids in a wide log *P* range. Applicability of the final EME-UHPLC-MS/MS method was demonstrated through validation and comparison of authentic samples with a pre-established routine method.

## Materials and methods

### Chemicals and reagents

Reference substances were purchased from two different vendors for the majority of analytes to prepare quality control samples independently from calibrators. Reference substances were purchased from the following companies: buprenorphine, codeine, codeine-d_3_, ethylmorphine, fentanyl, morphine, morphine-d_3_, O-desmethyl tramadol (O-DM-tramadol) and pethidine were from Lipomed (Arlesheim, Switzerland). Fentanyl, oxycodone, pethidine and tramadol were purchased from Sigma Aldrich (St. Louis, MA, USA). Codeine, cyclopropylfentanyl, ethylmorphine-d_5_, fentanyl-d_5_ methadone-d_3_, morphine, N-pyrrolidino etonitazene (etonitazepyne), methadone, oxycodone, oxycodone-d_6_ and tramadol-d_6_ were from Chiron (Trondheim, Norway). Buprenorphine-d_3_, ethylmorphine, ketobemidone and O-DM-tramadol were from Toronto Research Chemicals Inc. (TRC, Toronto, ON, Canada). Buprenorphine and methadone were purchased from Reckitt Benckiser (Hull, UK) and St. Olav University Hospital Pharmacy (Trondheim, Norway), respectively. Ketobemidone-d_3_ was from Alsachim (Strasbourg, France).

Bis(2-Ethylhexyl) phosphate (DEHP), bis(2-ethylhexyl) phosphite (DEHPi), 2-nitrophenyl octyl ether (NPOE) and ammonium formate (≥ 99.995% NH_4_HCO_2_, trace metal basis) were purchased from Sigma Aldrich. Thymol (Thy), 6-methylcoumarin (6MC), methanol (MeOH, LC-MS grade) and nitric acid (69% HNO_3_) were from Merck (Darmstadt, Germany). Ethyl acetate (EtOAc, HiPerSolv Chromanorm), n-heptane (HiPerSolv, Chromanorm) and sodium carbonate (Na_2_CO_3_) were purchased from VWR Chemicals (Leuven, Belgium). Formic acid (99.0% HCOOH, Optima LC-MS grade) was from Fischer Scientific (Leicestershire, UK). Type I water was obtained in-house using a Milli-Q purification system from Millipore (Molsheim, France). Quantisal extraction buffer was obtained from Immunalysis Corporation (Pomona, CA, USA).

### Preparation of solutions

#### Oral fluid samples

Oral fluid (OF) was sampled using Quantisal Oral Fluid Collection device from Immunalysis. A collector pad collected 1 mL (± 10%) OF specimen confirmed by the use of a colour indicator. The collector pad was subsequently submerged in a tube containing 3 mL Quantisal extraction buffer (Immunalysis). OF samples that arrive at the laboratory thus comprised OF diluted 1:3 (v/v) in the Quantisal buffer. Samples consisting of OF diluted in the Quantisal extraction buffer are henceforth referred to as “OF samples”. OF samples used in method development and validation were prepared by diluting drug-free OF with Quantisal buffer (1:3, v/v). Drug-free OF was donated anonymously by healthy volunteers employed at the Department of Clinical Pharmacology at St. Olav University Hospital (Trondheim).

External control samples of buprenorphine (*n* = 1), codeine (*n* = 3), methadone (*n* = 2) and morphine (*n* = 3) were obtained from LGC Proficiency Testing (Bury, UK). The samples consisted of OF spiked by the manufacturer and were diluted 1:3 (v/v) with Quantisal Extraction buffer prior to analysis.

#### Working solutions, calibrators, and quality controls

Stock solutions of target analytes were combined and diluted to working solutions in MeOH for each calibrator and quality control (QC) level. Calibrator and QC samples were prepared by spiking 2% working solutions in drug-free OF samples. Analytes were grouped in three calibration ranges, 0.1–5 μg/L, 1–50 μg/L and 3–50 μg/L, with four calibration concentrations per range. The calibration range corresponds to concentrations in pure OF. Calibrators and QC samples were prepared to account for dilution in the collection device. The internal standard (IS) working solution was prepared in MeOH:H_2_O (1:1, v/v). Working solutions, calibrators, QC samples and IS solution were stored at −20°C. Concentrations of target analytes and internal standards are further detailed in Table S2-3.

#### Deep eutectic solvent

The deep eutectic solvent was prepared by heating appropriate amounts of 6-methylcoumarin and thymol (1:2 molar ratio) at 70°C for 12 min. The solvent was vortex-mixed prior to every use.

### Electromembrane extraction

**Fig. 1:**
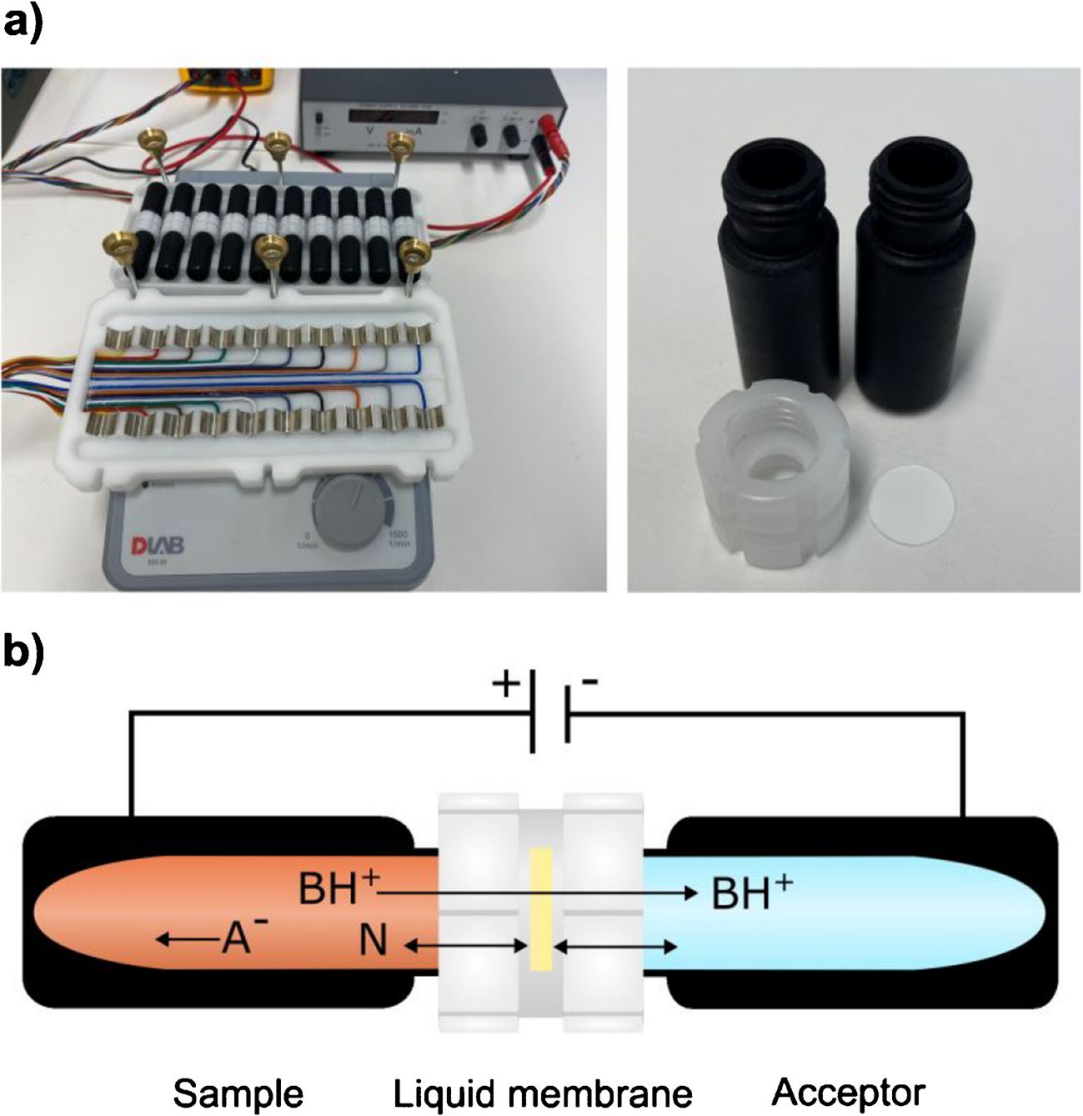
**a** Prototype conductive vial EME setup with ten EME cells (left). An EME cell consisted of conductive vials, a union, and PP support membrane (right). **b** EME of protonated bases (BH^+^). A^−^ is an anionic substance and N is a neutral molecule

EME was performed with a prototype device from Extraction Technologies Norway (Ski, Norway), shown in Fig. 1. The prototype utilized vials of a conductive polymer and has been described previously [27, 38]. Ten EME cells could operate simultaneously. Each cell comprised a sample vial, an acceptor vial and a flat polypropylene support membrane contained in a leak-tight union. The sample vial held the biological sample and sample diluent. The latter served to control pH in the sample. The acceptor vial contained the acceptor solution. The membrane was sandwiched between the union and one vial to prepare the liquid membrane. The liquid membrane was prepared by pipetting an organic solvent onto the PP support, while the union was mounted on a conductive vial. EME cells were assembled and placed on the device where they were subjected to horizontal agitation, and the electric field was coupled through the vials via electrodes in the device lid and an external power supply (ES 0300-0.45, Delta Elektronika BV, Zierikzee). Target analytes migrated from the sample (anode) through the liquid membrane contained in the union and into the acceptor (cathode). The acceptor vial was re-capped after extraction and transferred directly for UHPLC-MS/MS analysis of the acceptor solution.

The final extraction protocol was as follows. The sample vial held OF samples (100 μL), IS working solution (15 μL) and 0.1% (v/v) HCOOH (sample diluent, 175 μL). The liquid membrane was prepared by pipetting 8-μL membrane solvent (2:1 (v/v) mixture of NPOE and a deep eutectic solvent consisting of 6MC and Thy (1:2, molar ratio)) onto the support membrane (Accurel PP2E, 3M Deutschland GmbH, Wuppertal, Germany). A punched out silicone disk (2 mm thick, D 9 mm, I.D. 6 mm) was sandwiched between the union and membrane to provide electric insulation. The acceptor vial contained 300 μL 0.1% (v/v) HCOOH. EME cells were assembled, and extraction was carried out by applying 20 V for 20 min with agitation (875 rpm). Conditions during method development were the same as described above when not stated otherwise. Method development experiments were carried out using OF samples spiked to the middle of the calibration range of each analyte.

With the current prototype and liquid membrane, it was found that the membrane should be mounted on the donor vial, opposed to the acceptor vial, as recovery and precision of the most lipophilic compounds seemed to be affected by this detail. This has not been observed previously using other membrane solvents (e.g. NPOE) with conductive vial EME. The remaining target opioids were unaffected by the cell configuration. All reported recovery data (method development and validation) of opioids were obtained by mounting the membrane on the donor vial. However, some validation experiments were carried out with the opposite configuration.

Due to limited amount of prototype equipment, vials were washed and re-used. To avoid carry-over, vials were washed using the following procedure: (1) empty vials were filled with 1% HCOOH in MeOH (*t* > 12 h), and (2) vials were thoroughly rinsed with type I water followed by (3) MeOH.

### Liquid–liquid extraction — routine sample preparation method

The final EME method was compared to a routine method at St. Olav University Hospital (Trondheim, Norway) used to screen multiple drugs of abuse in OF, including ten of the target opioids (morphine, oxycodone, codeine, O-DM-tramadol, ethylmorphine, tramadol, ketobemidone, buprenorphine, fentanyl and methadone). The sample preparation was as follows. Automated liquid handling was performed by a Hamilton Microlab Star pipetting robot (Hamilton Company, Bonaduz, Switzerland). Calibrators and quality controls were prepared by the robot by spiking a mixture of Quantisal extraction buffer and type I water (1:3, v/v) with working solutions of analytes. Liquid–liquid extraction (LLE) was performed in a 2.2-mL 96-well plate (Irish Life Sciences, Longford, Ireland) by mixing samples (200 μL), IS working solution (25 μL), 0.2 M Na_2_CO_3_ (200 μL) and EtOAc/heptane (80:20, v/v, 1000 μL) for 1 min at 2100 rpm (Multi-format Plates & Tubes, Porvoir Sciences, Norfolk UK). The content was centrifuged for 5 min at 4235 rcf (Rotana 460, Hettich Zentrifugen, Tuttlingen, Germany). Aliquots of supernatant (800 μL) were transferred to a new 96-well collection plate. The supernatant was acidified with 0.1% HNO_3_ in MeOH (10 μL) and then evaporated under ambient air at 40 °C for 20 min using Ultravap sample concentrator (Porvair Sciences). Finally, samples were reconstituted in 60% MeOH in H_2_O (75 μL) and mixed (2100 rpm, 1 min). Chromatographic separation and detection were achieved using the UHPLC-MS/MS conditions described in the “UHPLC-MS/MS” section.

The method was validated prior to its implementation, and the following validation data were achieved for opioids: precision and accuracy at LLOQ CV ≤ 12% and bias ± 9%, intra-day CV ≤ 7%, inter-day CV ≤ 14%, bias ± 11%, ME (uncorrected) 55–102% (CV ≤ 10%), ME (corrected) 95–110 (CV ≤ 5%) and recoveries 13–86% (CV ≤ 15%).

### UHPLC-MS/MS

Target analytes were separated and detected by ultra-high-performance liquid chromatography–tandem mass spectrometry (UHPLC-MS/MS). Chromatographic conditions were similar for EME and LLE samples. Gradient elution was carried out at 60 °C on an Acquity UPLC BEH C_18_ (2.1 × 50 mm, 1.7 μm particles) analytical column with an Acquity UPLC BEH C_18_ (2.1 × 5 mm, 1.7 μm particles) pre-column on an Acquity UPLC I-Class instrument from Waters (Milford, MA, USA). Mobile phase A consisted of 5 mM NH_4_HCO_2_ (pH 10.1) and B consisted of MeOH. The flow rate was 0.5 mL/min and total run time 5.3 min. The gradient started with 5% B and continued to 30% B after 0.3 min, 50% at 2.7 min, 90% at 3.8 min and 98% at 4.8 min. The injection volume was 3 μL.

Analytes were detected using a Xevo TQ-S tandem mass spectrometer (Waters, Manchester, UK) equipped with a Z-spray electrospray interface. Positive ionization (ESI+) was performed in multiple reaction monitoring (MRM). The capillary voltage was 1 kV and ion source temperature 120 °C. The desolvation gas (nitrogen) was heated to 650 °C and delivered with a flow rate of 1000 L/h. Cone gas flow was 150 L/h. MRM transitions of analytes included in the EME-UHPLC-MS/MS protocol are provided in Table S4.

### Calibration and identification

Target opioids were identified by relative retention times and ion ratios between MRM transitions compared to calibrators. Weighted (1/×) quadratic calibration curves with four calibration levels, excluding the origin, were used based on analyte peak area normalized to IS peak area. Calculated analyte concentrations related to the concentration in OF, accounting for dilution in the collection buffer.

### Validation

The method was validated based on guidelines given by Peters et al. [39] and the European Medical Agency (EMA) [40] and included linearity, precision, accuracy, extraction recovery, matrix effects, post-extraction stability and carry-over.

Linearity was evaluated by assessing the correlation coefficient (R) when applying a 1/× weighted linear calibration model with three replicates per calibrator. The lower limit of quantification (LLOQ) was set as the lowest calibrator concentration. Requirements of LLOQ was a signal-to-noise (S/N) above ten and three for the quantifying and qualifying ion, respectively, and inter-day CV and accuracy within ± 20%. Precision and accuracy at LLOQ were evaluated by analysing one spiked QC sample on ten days.

The method precision and accuracy were assessed at three QC levels. The intra- and inter-day precision at each QC level was evaluated by analysis of six parallels on 1 day and one sample at three QC concentrations on 10 different days, respectively. Accuracy was determined using inter-day precision data and by analysis of external control samples of selected analytes (morphine, buprenorphine, codeine and methadone). *Z*-scores were calculated as the difference between our measured value and assigned value (mean of laboratories included in the proficiency testing programme), divided by the standard deviation for proficiency assessment (SDPA).

Extraction recovery (RE) and matrix effects (ME) were calculated by the following equations:

1 $$RE=\frac{R_{\textrm{pre}}}{R_{\textrm{post}}}\times 100\%$$

2 $$ME=\frac{A_{\textrm{post}}}{A_{\textrm{ref}}}\times 100\%$$

where *R*_pre_ and *R*_post_ correspond to the IS-normalized analyte peak area in OF samples spiked with analytes pre- and post-extraction, respectively, with the internal standard added post-extraction in both cases. *A*_post_ *and A*_ref_ correspond to the analyte peak areas in spiked blank extracts and a neat acceptor, respectively. The recovery was evaluated at the high and low QC level with six parallels per concentration. Recovery was related to the EME step and did not include recovery from the OF collector pad. Matrix effects were assessed with six OF samples from different individuals at the high and low QC level.

Carry-over was evaluated by inspecting chromatograms of analyte-free OF samples after injection of a sample five times the concentration of the highest calibrator. Post-extraction stability was assessed by reinjection of QC samples (*n* = 5 per level) and calibrators left on the auto-sampler (10°C) for 1, 3 and 7 days.

### Method comparison with reference method

To demonstrate the applicability of the method, authentic anonymized OF samples were analysed, and results were compared to a routine screening method at the Department of Clinical Pharmacology at St. Olavs hospital (Trondheim, Norway). Samples (*n* = 30) that had been subjected to drug testing were anonymized and stored for 4 weeks at 4°C and up to 1 week at −20°C. The samples were reanalysed with EME-UHPLC-MS/MS and a routine method that employed LLE-UHPLC-MS/MS. The collected samples of methadone and buprenorphine were above the highest calibrator level and were diluted with a water/Quantisal buffer mixture (1:3 v/v) prior to analysis with EME and the routine method. The routine screening method targeted a total of 29 drugs of abuse, including amphetamine-type and other stimulants, benzodiazepines, hallucinogens, Z-hypnotics and opioids. Ten of the target opioids (morphine, oxycodone, codeine, O-DM-tramadol, ethylmorphine, tramadol, ketobemidone, buprenorphine, fentanyl and methadone) were included in both methods. Results were compared using Passing and Bablok regression [41] with MedCalc Statistical Software version 20 (MedCalc Software Ltd., Ostend, Belgium). According to the Regional Committee of Research Ethics, no formal approval is needed for a brief presentation of routine results as part of a methodological article.

### Characterization of the liquid membrane

The final membrane solvent was subjected to a characterization aiming to define the application range in terms of analyte log *P* values. Experimental details and interpretations were the same as in previous work [37]. In short, based on extraction of 90 basic model substances, the extraction window of a liquid membrane was expressed through analyte log *P* values, from log *P*_low_ to log *P*_high_. Herein, log *P*_low_ is the lowest log *P* value where RE ≥ 40% is achieved for ≥ 50% of analytes in the range from log *P*_low_ to log *P*_low_ +0.5. Similarly, ≥ 50% of model analytes in the log *P* range from log *P*_high_ – 0.5 to log *P*_high_ are extracted with RE ≥ 40%.

## Results and discussion

### Method development

#### Selection of the liquid membrane

Selecting an appropriate membrane solvent is a key step for efficient and robust EME. Attention should be on the extraction recoveries, repeatability and system stability. The latter is assessed by monitoring the extraction current. Although usually found by trial and error, efforts have been put forward to study the application range of known membrane solvents to guide method development. Here, the *extraction window* of a liquid membrane is defined as the range of analyte log *P* values where high recoveries are expected [37]. For basic analytes, NPOE has been established as the first choice based on stability, low current and high reproducibility. The extraction window of NPOE was recently defined as 2 < log *P* < 6 [37]. Compounds with log *P* < 2 are likely too hydrophilic for efficient transfer into NPOE. On the other side, very lipophilic compounds have unfavourable partitioning coefficient between the liquid membrane and acceptor and are trapped in the liquid membrane.

**Fig. 2:**
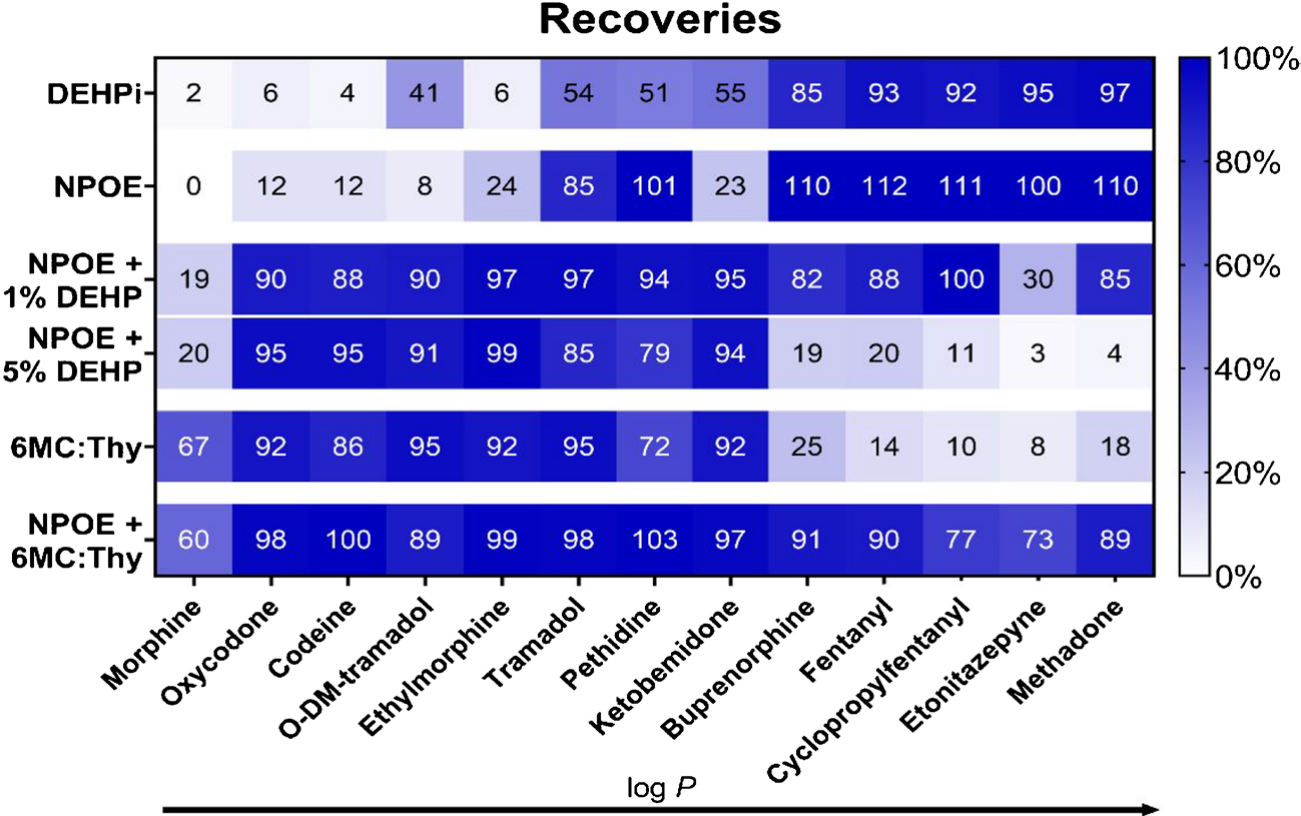
Recoveries of target opioids with six membrane solvents: DEHPi, NPOE, and NPOE with 1% and 5% (w/w) DEHP, 6MC:Thy (1:2, molar ratio), and a 1:2 (v/v) mixture of 6MC:Thy (1:2, molar ratio) and NPOE. Applied voltage was 50 V for all membranes except for 6MC:Thy (1:2, molar ratio) (10 V). t = 20 min

The target opioids were extracted from OF samples for 20 min with six membrane solvents. Recoveries are presented in Fig. 2. Analytes are ordered by their predicted log *P* values, which ranged from 0.7 (morphine) to 5.0 (methadone) (Table S1). As expected from the extraction window of NPOE (2 < log *P* < 6), opioids in the lower log *P* range were not efficiently extracted with this membrane solvent. For the other membrane solvents, extraction windows were not clearly defined at this point.

DEHPi has been reported for extraction of hydrophilic analytes [42], and this solvent was tested as an alternative to NPOE. In the present experiments, extractions with DEHPi was generally only efficient for the most lipophilic opioids (log *P* > 2.5), similarly to NPOE, with the exception of O-DM-tramadol (log *P* = 1.6), which was extracted with medium efficiency (41%).

Addition of the ionic carrier DEHP to NPOE is a well-established measure to increase extraction efficiency of hydrophilic analytes [43, 44]. As seen from Fig. 2, adding 1% DEHP to NPOE allowed exhaustive extraction of 11 out of 13 target opioids and improved mass transfer of all compounds in the lower end of the log *P* range. However, morphine recoveries were unsatisfactory (RE 19%, CV 27%). Increasing the amount of carrier to 5% did not improve morphine recoveries and resulted in membrane trapping of the most lipophilic compounds. Mass transfer of morphine was not improved upon increasing the extraction potential or time or upon altering the pH (data not shown).

Deep eutectic solvents have emerged as alternative extraction solvents due to their greenness, low-cost and applicability for a wide range of solutes [45]. A eutectic mixture is a liquid comprising of two or more components that interact through hydrogen bonds resulting in melting point depression relative to individual components. Hydrophobic deep eutectic solvents have been used as membrane solvents in EME previously [46–49]. In particular, eutectic mixtures of coumarin and thymol (Thy) have demonstrated efficient extraction of compounds in a log *P* range similar to the target opioids [47, 49]. In the present experiments, analytes were first extracted with a deep eutectic solvent consisting of 6-methylcoumarin (6MC) and Thy in a molar ratio of 1:2, which was previously used in extraction of methotrexate and metabolites (−2.2 < log *P* < 0.9) from plasma [48]. 6MC was used opposed to coumarin due to lower water solubility making the liquid membrane inherently more stable. Despite this, the deep eutectic solvent was less stable than the other membrane solvents and had to be operated at lower voltage (10 V) to avoid excessive current and attain system stability. Extraction with 6MC:Thy (1:2, molar ratio) covered the lower log *P* range of target opioids, with satisfactory results for morphine (RE 67%, CV 10%). Recoveries were however low for the most lipophilic opioids. Although some lipophilic analytes have been extracted with coumarin and Thy in literature, the higher intrinsic hydrophobicity of 6MC could explain the unexpectedly low recoveries of buprenorphine, fentanyl, cyclopropylfentanyl, etonitazepyne and methadone the present experiment.

In a final set of experiments, the deep eutectic solvent was mixed with NPOE. The solvents were mixed in a 1:2 (v/v) ratio of 6MC:Thy (1:2, molar ratio) and NPOE. The membrane solvent consisting of 6MC, Thy and NPOE covered all target opioids, with high recoveries and acceptable precision (CV ≤ 18%) for all analytes. In addition, stability was improved compared to pure 6MC:Thy (1:2, molar ratio), and stable extractions could be carried out at higher voltages.

#### Extraction window of the liquid membrane

**Fig. 3:**
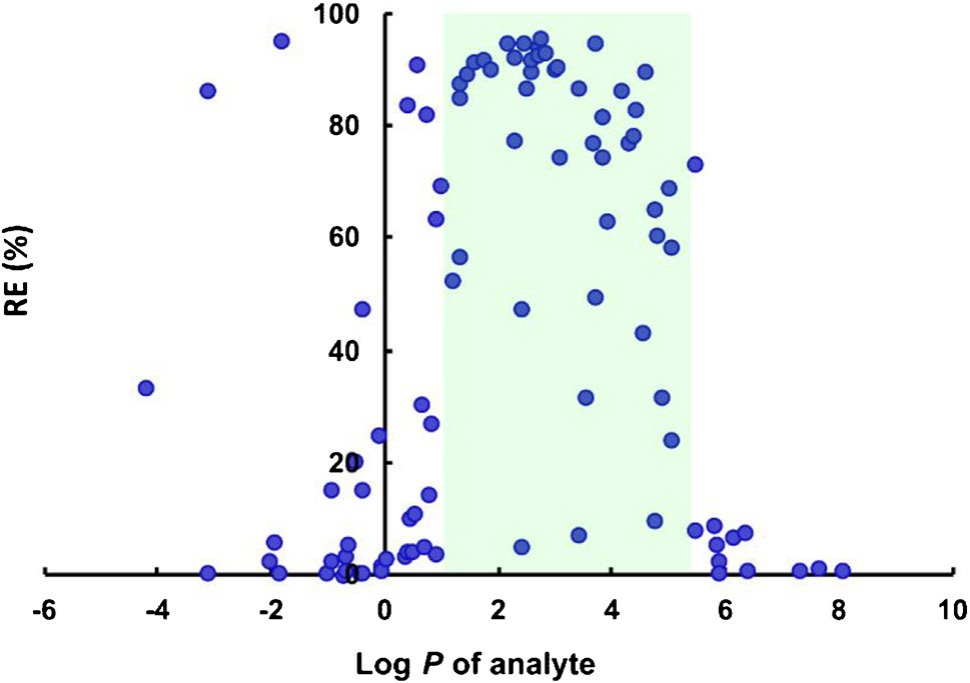
Extraction recovery versus log *P* for 90 bases using a 1:2 (v/v) mixture of 6MC:Thy (1:2, molar ratio) and NPOE as the liquid membrane. The extraction window is highlighted

The selected liquid membrane was further characterized by studying its extraction window. The extraction window of the membrane was determined by the same procedure described by Zhou et al. [37], where 90 basic model analytes were extracted from pseudo samples using conductive vial EME. The extraction window is here expressed through analyte log *P* values. Figure 3 shows the recoveries of model analytes plotted against their predicted log *P* values. The extraction window of the 1:2 (v/v) mixture 6MC:Thy (1:2, molar ratio) and NPOE was found to be 1 < log *P* < 5.5. By comparison to pure NPOE (2 < log *P* < 6), the extraction window was shifted slightly towards more polar compounds, and this allowed simultaneous extraction of all target opioids.

#### Sample diluent, acceptor, extraction potential, and time

After selecting a mixture of 6MC, Thy and NPOE as the liquid membrane, further method development included selection of sample diluent and acceptor, extraction potential and time. The agitation rate was set to 875 rpm based on previous experience.

**Fig. 4:**
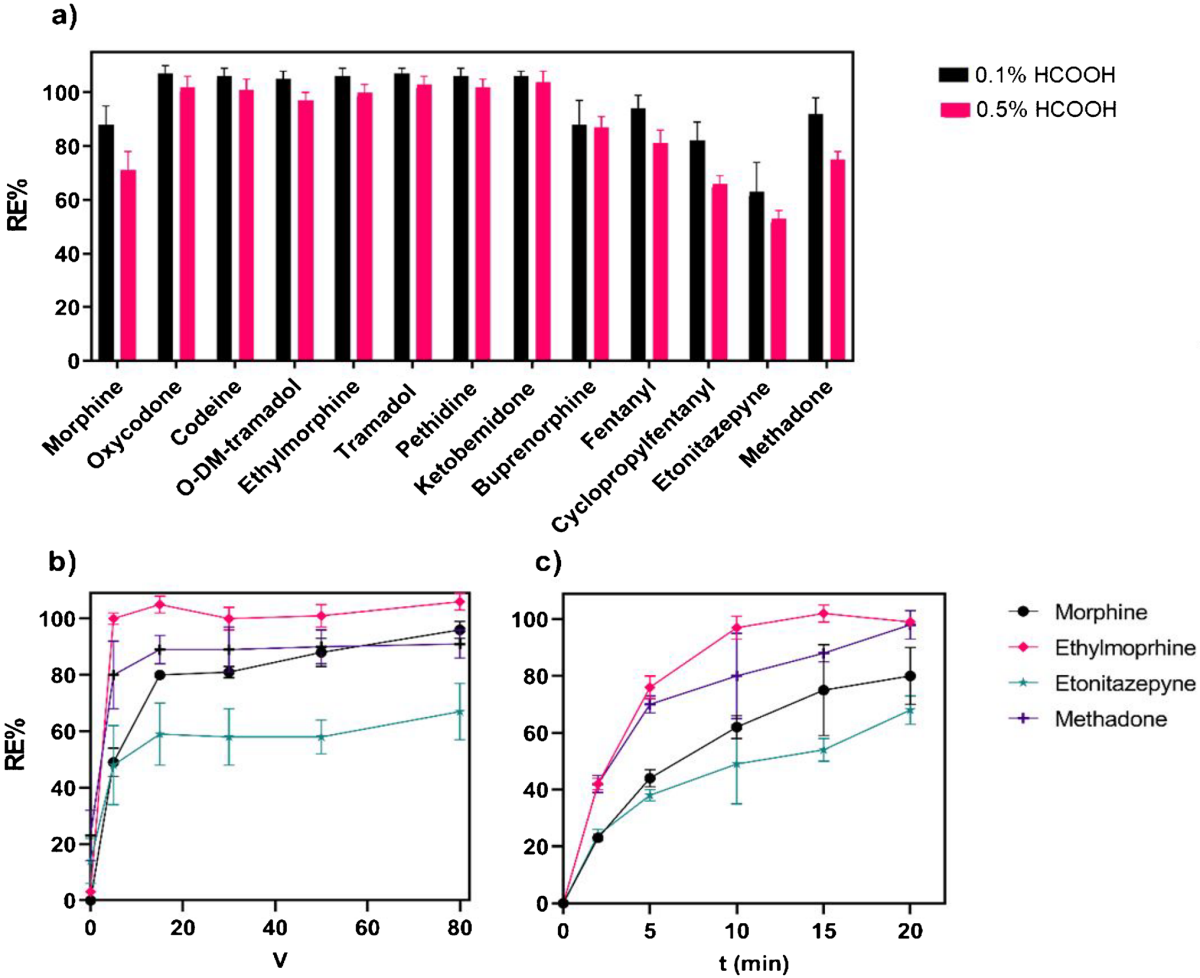
Operational parameters for EME of opioids from OF samples using a mixture of 6MC, Thy, and NPOE as membrane solvent. ***a*** Recovery using two concentrations of HCOOH as sample diluent and acceptor (V = 50 V, t = 20 min). ***b*** Recovery with varying extraction potential selected opioids (t = 20 min). ***c*** Recovery as a function of extraction time for selected opioids (V = 20 V)

Solutions of 0.1% and 0.5% (v/v) HCOOH were evaluated as sample diluent and acceptor (Fig. 4a). Although not always possible, choosing the same reagent as the sample diluent and acceptor was considered advantageous in relation to operational simplicity. OF samples were diluted 1:2 (v/v) with the sample diluent, resulting in a sample pH of 5 and 3 for 0.1% and 0.5% HCOOH, respectively. The acceptor pH was 2.7 and 2.5, respectively. After applying 50 V for 20 min, both conditions resulted in high performance with an average recovery for all analytes of 96% and 88% for 0.1% and 0.5% HCOOH, respectively. The 0.1% HCOOH solution was selected as the final sample diluent and acceptor.

The extraction potential was selected by studying analyte recovery when applying 0, 5, 15, 30, 50 and 80 V for 20 min. Based on the recorded system current (Fig. S1), the system was considered stable with all conditions. Figure 4b show the extraction recovery with varying extraction potential for selected opioids. The optimal extraction potential was compound dependent, and 20 V was selected as a compromise. Method development data for all opioids are presented in Table S5-6.

The final extraction time was selected to be 20 min as a compromise between recovery, precision and sample throughput. Figure 4c shows recovery after extraction for 2, 5, 10 and 20 min for selected opioids. All compounds were exhaustively extracted after 20 min with the exception of morphine and etonitazepyne, which reached recoveries of 80% and 68% respectively. The precision was acceptable with CV ≤ 15%.

### Validation

The method was validated according to guidelines by Peters et al. and the European Medical Agency [39, 40]. Linearity, LLOQ, intra-day precision, inter-day precision and bias, recoveries, matrix effects, carry-over and post-extraction stability were assessed. A summary of validation data is shown in Table 1.

**Table 1:** Validation data of opioids: coefficient of correlation (*R*), precision (CV%), and accuracy (bias%) at the lower limit of quantification (LLOQ) and three QC levels, recoveries (RE), matrix effects (ME), and internal standard corrected matrix effects. The quantification range was from LLOQ to 50 × LLOQ for all analytes

Analyte, QC concentration (μg/L)	LLOQ (μg/L)	Intra-day CV (%)	Inter-day CV (%)	Bias (%)	RE (CV%)	ME (CV%)	IS corrected ME (CV%)	Linearity (R)
		*n* = 6	*n* = 10	*n* = 10	*n* = 6	*n* = 6	*n* = 6	
Morphine	3		3	−0.2				0.9996
4.5		2	2	−0.8	66 (14)	98 (3)	102 (2)	
30		2	3	5				
120		1	2	8	85 (4)	97 (3)	101 (1)	
Oxycodone	1		4	−8				0.9998
1.5		2	3	−9	104 (3)	97 (3)	102 (2)	
10		1	2	−6				
40		1	1	−2	97 (3)	96 (3)	98 (2)	
Codeine	1		6	−6				0.9996
1.5		5	5	−8	97 (2)	95 (3)	103 (1)	
10		3	5	0.2				
40		2	9	−2	102 (3)	94 (3)	103 (1)	
O-DM-tramadol	1		5	−11				0.9996
1.5		2	8	−6	96 (4)	97 (3)	104 (2)	
10		3	5	−4				
40		2	2	−5	102 (2)	97 (3)	105 (2)	
Ethylmorphine	1		3	−8				0.9998
1.5		2	2	−8	97 (2)	96 (3)	100 (2)	
10		1	3	−3				
40		1	3	−2	100 (2)	96 (3)	99 (2)	
Tramadol	3		2	−12				0.9998
4.5		2	5	−11	99 (1)	93 (4)	97 (2)	
30		2	4	−9				
120		2	4	−6	99 (1)	99 (4)	103 (2)	
Pethidine	1		8	−4				0.9982
1.5		3	4	−5	101 (1)	97 (3)	100 (2)	
10		2	4	−2				
40		3	4	0.8	98 (2)	99 (3)	102 (2)	
Ketobemidone	1		4	−5				0.9998
1.5		3	4	−6	98 (3)	96 (4)	102 (2)	
10		2	3	−3				
40		2	4	−1	102 (4)	92 (2)	99 (1)	
Buprenorphine	3		2	−2				0.9994
4.5		4	4	−2	79 (7)	212 (1)	100 (2)	
30		2	3	−1				
120		2	3	−0.4	80 (6)	180 (4)	97 (1)	
Fentanyl	0.1		13	−9				0.9993
0.15		2	8	−11	62 (13)	105 (4)	98 (4)	
1		2	5	−8				
4		2	3	−9	69 (14)	104 (4)	98 (2)	
Cyclopropylfentanyl	0.1		16	−8				0.9954
0.15		7	11	−5	54 (18)	113 (3)	88 (2)	
1		11	9	−6				
4		6	12	−2	60 (20)	108 (4)	89 (1)	
*Etonitazepyne	0.1		45	−16				0.9663
0.15		10	27	−12	39 (23)	127 (4)	99 (5)	
1		28	25	−20				
4		34	25	−17	45 (21)	116 (4)	96 (2)	
Methadone	3		6	−2				0.9995
4.5		2	3	−4	66 (19)	133 (3)	104 (3)	
30		1	2	−1				
120		2	3	0.2	66 (18)	122 (3)	101 (2)	

*Validation criteria not fulfilled

Etonitazepyne could not be quantified with acceptable precision (CV 25–34%) and accuracy (−20%). This is further discussed in the “Extraction recoveries and the effect on precision” section. Validation results of etonitazepyne are included in Table 1 but are excluded from the result and discussion below.

Calibration curves were linear in the calibration range with correlation coefficients *R* ≤ 0.9954. The precision and accuracy at LLOQ were acceptable (CV ≤ 16%, bias ± 16%). The intra-day CV was ≤ 11%, inter-day CV ≤ 12% and bias within ± 11%, which was also considered satisfactory. External control samples of buprenorphine, codeine, methadone and morphine were quantified with *Z*-scores in the range −1.5 ≤ Z ≤ 1.7, which was within requirements (|*Z*| ≤ 2.0) and indicated good accuracy.

Matrix effects ranged from 93 to 133% (CV ≤ 4%) for all analytes except buprenorphine, which had matrix effects of 212% and 180% (CV ≤ 4%) at the low and high concentration, respectively. The matrix effects were 88–105% (CV ≤ 4%) for all analytes when corrected with internal standards. In an additional experiment using a reference solution (neat acceptor, Eq. 2) spiked with a drop of membrane solvent, the calculated matrix effects of buprenorphine were 100–103% (CV ≤ 4%), which suggests that the apparent signal enhancement found in validation was likely attributed to the presence of liquid membrane solvent in the acceptor.

Analytes were stable in extracts on the auto-sampler (10 °C) for at least seven days. Carry-over after injection of a sample five times the concentration of the highest calibrator was acceptable (≤ 20% of LLOQ). However, OF concentrations of buprenorphine may occasionally be much higher if sampling occurs shortly after intake [50], and this must be considered in a routine setting.

Regarding the quantitative data, recovery from the collector swab in the Quantisal Collection Device was not evaluated. Previous studies have found the recovery from the collection device to be high for opioids [51, 52].

#### Extraction recoveries and the effect on precision

Efficient extraction of all target opioids was achieved with extraction recoveries in the range from 39 to 104%. Corresponding CVs were ≤ 20% for all analytes except for etonitazepyne (CV = 23%) (Table 1). Analytes that were regarded as moderately lipophilic (1 < log *P* < 2.5) had recoveries above 96% with low variation (CV ≤ 4%), whereas morphine (log *P* = 0.7) was extracted with recovery above 66% with CV ≤ 14%. Recovery of lipophilic analytes (log *P* > 2.5) was in the range 39–80% with CVs 6–23%. In general, recoveries of lipophilic analytes (i.e. buprenorphine, fentanyl, cyclopropylfentanyl, etonitazepyne and methadone) were associated with slightly higher variation compared to other analytes in the method and compared to similar analytes extracted with NPOE previously [27]. The lipophilic analytes ultimately required more correction by internal standards than the remaining target opioids. Validation data were satisfactory for buprenorphine, fentanyl and methadone, which all had deuterated analogues to compensate variability. For cyclopropylfentanyl and etonitazepyne, no deuterated standards were available, and methadone-d_3_ was used. Validation data complied with guideline requirements for cyclopropylfentanyl, while etonitazepyne suffered in terms of linearity and precision and would likely benefit from having a dedicated internal standard.

#### Application and comparison with routine method

Anonymized positive OF samples for buprenorphine (*n* = 13), codeine (*n* = 4), ketobemidone (*n* = 3), morphine (*n* = 2) and methadone (*n* = 9) from a routine screening method were subjected to EME to demonstrate method applicability. The sample preparation of the routine screening method consisted of LLE, evaporation and reconstitution.

**Fig. 5:**
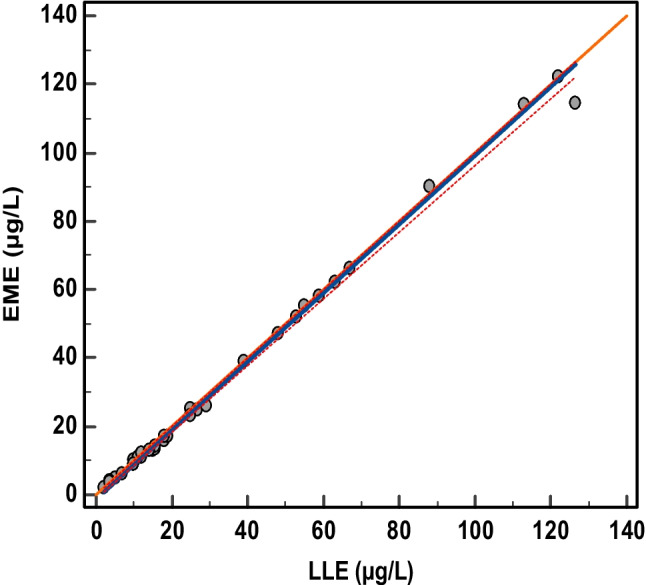
Passing and Bablok regression between EME-UHPLC-MS/MS and LLE-UHPLC-MS/MS. The *Y*-intercept was −1.00 (95% CI from [−1.0000, 0.1389]), and the slope was 1.00 (95% CI [0.9722, 1.000]). The Spearman rank correlation coefficient was 0.998 (95% CI [0.996, 0.999])

The correspondence between methods was within ± 15% for 32 of the 33 concentration pairs. One sample of codeine deviated by 23%, which was considered acceptable. The Passing and Bablok regression line (Fig. 5) between EME and LLE was [EME] = 1[LLE] − 1. The 95% confidence intervals of the slope ([0.97, 1.00]) and intercept ([−1.00, 0.14]) contained one and zero, respectively, which implied no systematic or proportional differences between methods.

Compared to the LLE protocol, EME involved fewer steps with a significant reduction in organic solvent consumption, which made EME more favourable with respect to principles of green analytical chemistry [53, 54]. We recently demonstrated this in another work by comparing greenness scores of conductive vial EME and an LLE method similar to the routine OF screening method [38].

## Conclusion

In the present contribution, selected opioids were successfully extracted from OF samples using conductive vial EME. Simultaneous EME of analytes in a wide log *P* range is a challenge, as narrow extraction windows of many liquid membranes cause discrimination of either lipophilic or hydrophilic analytes. By adding 6-methyl coumarin and thymol to 2-nitrophenyl octyl ether, the extraction window was shifted slightly towards more polar substances, and this allowed simultaneous extraction of all target opioids. When EME was combined with UHPLC-MS/MS, validation data were within recommended guidelines for 12 out of 13 target opioids. In general, the most lipophilic analytes were prone to higher variability than the remaining analytes; however, quantitative results were satisfactory when applying appropriate internal standards. External control samples containing both hydrophilic opioids (morphine, codeine) and lipophilic opioids (buprenorphine, methadone) had satisfactory *z*-scores (|*Z*| ≤ 2.0). In addition, quantitative data of authentic OF samples were in accordance with a reference routine method that used conventional sample preparation. The presented data thus illustrate the applicability of conductive vial EME, which can be a greener alternative to conventional sample preparation in the future. Lastly, the results further motivate research on the application ranges and characterization of liquid membranes in EME, which will be useful for future method developers.

## Supplementary information

ESM 1 (PDF 625 kb)
