# Supplementary material for: Conductive vial electromembrane extraction of opioids from oral fluid
Source: Anal Bioanal Chem. 2023 Jun 29;415(22):5323–35. doi: 10.1007/s00216-023-04807-3 (PMC10444644; doi:10.1007/s00216-023-04807-3)
Supplement: Supplementary file 1 — (PDF 625 kb) [file 216_2023_4807_MOESM1_ESM.pdf]

Supplementary information for

Conductive vial electromembrane extraction of opioids from oral fluid

Tonje Gottenberg Skaalvik[a,b], Chen Zhou[b,c], Roger Trones[d], Elisabeth Leere Øiestad[b,e], Solfrid Hegstad[a], Stig Pedersen-Bjergaard[b,f]

[a] Department of Clinical Pharmacology, St. Olav University Hospital, Professor Brochs Gate 6, 7030 Trondheim

[b] Department of Pharmacy, University of Oslo, P.O. Box 1068 Blindern, 0316 Oslo, Norway

[c] West China School of Public Health and West China Fourth Hospital, Sichuan University, Chengdu, 610041, China

[d] Extraction Technologies Norway, Verkstedveien 29, 1424 Ski, Norway

[e] Department of Forensic Sciences, Division of Laboratory Medicine, Oslo University Hospital, P.O. Box 4459 Nydalen, 0424, Oslo Norway

[f] Department of Pharmacy, Faculty of Health and Medical Sciences, University of Copenhagen, Universitetsparken 2, 2100 Copenhagen, Denmark

Content

1 Target opioids 2
2 Concentrations 4
3 UHPLC-MS/MS 5
4 Method development data 6
4.1 Effect on voltage and time 6
4.2 Extraction current 7

1 Target opioids

Structure and chemical properties (pKa, log *P* and log $D_{pH=5}$) of target analytes are listed in Table S1. Properties were calculated using chemicalize.com (Chemaxon, 2023-01-24).

Table S1: Names, structure, predicted pKa (basic), log P and log $D_{pH=5}$ of target opioids

Analyte	Formula	Structure	pKa (basic)	Log *P*	Log $D_{pH\ 5}$
Morphine	$C_{17}H_{19}NO_3$	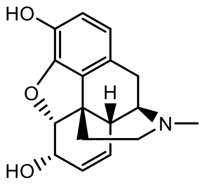	8.65	0.72	-2.20
Oxycodone	$C_{18}H_{21}NO_4$	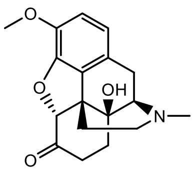	7.77	1.04	-1.66
Codeine	$C_{18}H_{21}NO_3$	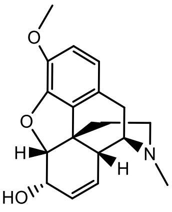	8.89	1.34	-2.01
O-Desmethyltramadol (O-DM-tramadol)	$C_{15}H_{23}NO_2$	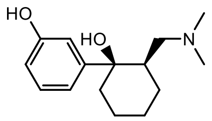	9.69	1.62	-1.14
Ethylmorphine	$C_{19}H_{23}NO_3$	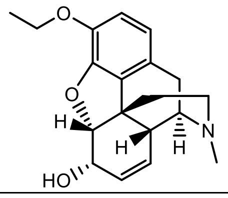	8.89	1.70	-1.65
Tramadol	$H_{16}H_{25}NO_2$	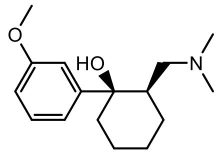	9.38	2.45	-1.00
Pethidine	$C_{15}H_{21}NO_2$	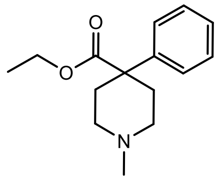	8.16	2.47	-0.54

Analyte	Formula	Structure	pKa (basic)	Log *P*	Log $D_{pH\ 5}$
Ketobemidone	$C_{15}H_{21}NO_2$	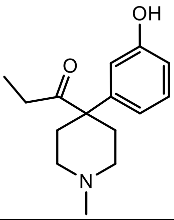	8.09	2.49	-0.28
Buprenorphine	$C_{29}H_{41}NO_4$	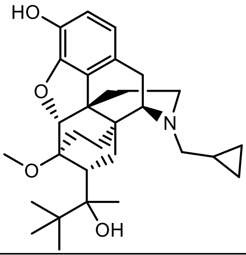	10.11	3.03	0.55
Fentanyl	$C_{22}H_{28}N_2O$	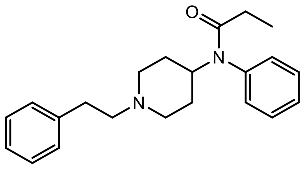	8.47	3.82	0.63
Cyclopropylfentanyl	$C_{23}H_{28}N_2O$	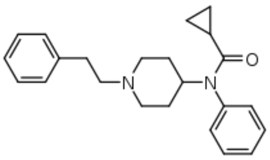	8.47	3.90	0.71
Etonitazepyne (N-Pyrrolidino Etonitazene)	$C_{22}H_{26}N_4O_3$	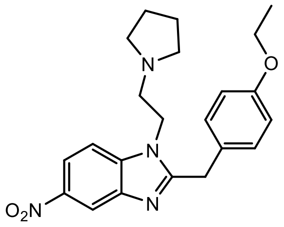	9.11	4.00	0.59
Methadone	$C_{21}H_{27}NO$	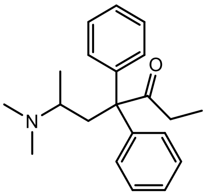	9.12	5.00	1.60

2 Concentrations

Calibrator and quality control (QC) levels of target analytes, and internal standard concentrations are detailed in Table S2 and Table S3. The concentrations given in Table S2 refer to concentration in oral fluid, whereas calibrator and QC samples were prepared with an additional 1:3 dilution corresponding to the dilution occurring in the Quantisal OF collection device.

Table S2 Calibrator (Std) and quality control (QC) concentrations of target analytes. Values correspond to concentration in OF, and the Std and QC samples were prepared with an additional dilution (1:3) as oral fluid is diluted in the Quantisal collection device.

**Analyte**	**Std1 (µg/L)**	**Std2 (µg/L)**	**Std3 (µg/L)**	**Std4 (µg/L)**	**LLOQ QC (µg/L)**	**QC1 (µg/L)**	**QC2 (µg/L)**	**QC3 (µg/L)**
Morphine	3	15	45	150	3	4.5	30	120
Oxycodone	1	5	15	50	1	1.5	10	40
Codeine	1	5	15	50	1	1.5	10	40
O-DM-tramadol	1	5	15	50	1	1.5	10	40
Ethylmorphine	1	5	15	50	1	1.5	10	40
Tramadol	3	15	45	150	3	4.5	30	120
Pethidine	1	5	15	50	1	1.5	10	40
Ketobemidone	1	5	15	50	1	1.5	10	40
Buprenorphine	1	5	15	50	1	1.5	10	40
Fentanyl	0.1	0.5	1.5	5	0.1	0.15	1	4
Cyclopropylfentanyl	0.1	0.5	1.5	5	0.1	0.15	1	4
Etonitazepyne	0.1	0.5	1.5	5	0.1	0.15	1	4
Methadone	3	15	45	150	3	4.5	30	120

Table S3: Concentration of isotopically labelled internal standards in IS- working solution (prepared in MeOH/H_2O 50:50)

**Compound**	**Concentration (µg/L)**
Morphine-d_3	50
Oxycodone-d_6	10
Codeine-d_3	10
Ethylmorphine-d_5	40
Ketobemidone-d_3	10
Tramadol-d_6	10
Buprenorphine-d_3	50
Fentanyl-d_5	2
Methadone-d_3	5

3 UHPLC-MS/MS

MRM transitions, cone voltage, collision energy and retention times of target analytes and internal standards are listed in Table S4.

*Table S4: MRM transitions, cone voltage, collision energies and retention times of target analytes and internal standards. * denotes the quantifying MRM transition.*

Compound	MRM transition (*m/z*)	Cone voltage (V)	Collision energy (eV)	RT (min)	IS
Morphine	286 > 201*<br>286 > 165	50<br>50	25<br>40	1.43	Morphine-d_3
Oxycodone	316 > 241*<br>316 > 256	50<br>50	25<br>25	2.64	Oxycodone-d_6
Codeine	300 > 215*<br>300 > 165	50<br>50	25<br>40	2.23	Codeine-d_3
O-DM tramadol	250 > 58*	30	14	2.47	Ketobemidone-d_3
Ethylmorphine	314 > 229*<br>314 > 165	50<br>50	25<br>40	2.88	Ethylmorphine-d_5
Ketobemidone	248 > 230*<br>248 > 70*	68<br>68	20<br>30	2.16	Ketobemidone-d_3
Pethidine	248 > 70*<br>248 > 174	90<br>90	40<br>30	3.43	Tramadol-d_6
Tramadol	264 > 58*	50	40	3.51	Tramadol-d_6
Buprenorphine	468 > 396*<br>468 > 414	60<br>60	42<br>36	4.33	Buprenorphine-d_3
Fentanyl	337 > 188*<br>337 > 105	54<br>54	22<br>32	3.91	Fentanyl-d_5
Cyclopropylfentanyl	349 > 188*<br>349 > 105	40<br>40	22<br>40	3.96	Methadone-d_3
Etonitazepyne	395 > 98*	26	22	3.85	Methadone-d_3
Methadone	310 > 223*<br>310 > 105	50<br>50	18<br>60	4.06	Methadone-d_3
Morphine-d_3	289 > 201	50	25	1.41	
Oxycodone-d_6	322 > 247	50	25	2.60	
Codeine-d_3	303 > 215	40	24	2.21	
Ethylmorphine-d_5	319 > 165	50	25	2.85	
Ketobemidone-d_3	251 > 70	68	30	2.15	
Tramadol-d_6	270 > 64	50	40	3.48	
Fentanyl-d_5	342 > 188	54	22	3.91	
Buprenorphine-d_3	471 > 396	60	42	4.33	
Methadone-d_3	313 > 268	24	14	4.05	

4 Method development data

4.1 Effect on voltage and time

Recovery of target opioids from OF samples with varying voltage and time are presented in Table S5 and S6, respectively. The sample comprised OF sample diluted 1:2 (v/v) with 0.1% HCOOH, and the acceptor was 0.1% HCOOH. The liquid membrane was a 1:2 (v/v) mixture of 2-nitrophenyl octyl ether (NPOE) and a deep eutectic solvent consisting of 6-methylcoumarin (6MC) and thymol (Thy) (1:2, molar ratio).

Table S5 Extraction recovery (RE) and coefficient of variance (CV) of target opioids from OF samples with varying extraction potential (V). Liquid membrane = 6MC:Thy (1:2, molar ratio) + NPOE (1:2, v/v), t = 20 min, n = 4.

	RE (CV%)					
**Analyte**	**0 V**	**5 V**	**15 V**	**30 V**	**50 V**	**80 V**
Morphine	0 (3)	49 (11)	80 (1)	81 (3)	88 (5)	96 (4)
Oxycodone	1 (13)	100 (3)	103 (6)	103 (3)	99 (3)	107 (2)
Codeine	2 (16)	101 (1)	102 (4)	104 (3)	103 (3)	108 (2)
O-DM-tramadol	0 (7)	95 (1)	100 (5)	96 (3)	98 (4)	101 (2)
Ethylmorphine	3 (48)	100 (2)	105 (2)	100 (4)	101 (4)	106 (3)
Tramadol	11 (14)	101 (1)	101 (1)	98 (3)	97 (5)	100 (0.5)
Pethidine	28 (6)	98 (4)	99 (2)	96 (3)	95 (3)	98 (1)
Ketobemidone	2 (14)	102 (1)	104 (1)	102 (3)	100 (3)	104 (1)
Buprenorphine	33 (16)	84 (9)	89 (4)	87 (7)	87 (7)	87 (6)
Fentanyl	27 (31)	77 (16)	86 (8)	87 (6)	85 (7)	87 (6)
Cyclopropylfentanyl	20 (41)	69 (21)	79 (12)	81 (10)	82 (6)	83 (10)
Etonitazepyne	14 (55)	48 (29)	59 (19)	58 (20)	58 (10)	67 (15)
Methadone	23 (39)	80 (15)	89 (6)	89 (10)	90 (7)	91 (6)

Table S6 Extraction recovery (RE) and coefficient of variance (CV) of target opioids from OF samples with varying extraction time (min). Liquid membrane = 6MC:Thy (1:2, molar ratio) + NPOE (1:2, v/v), V = 20 V, n = 4

	RE (CV%)				
**Analyte**	**2 min**	**5 min**	**10 min**	**15 min**	**20 min**
Morphine	23 (6)	44 (8)	62 (7)	75 (21)	80 (12)
Oxycodone	39 (7)	73 (5	96 (5)	101 (5)	104 (2)
Codeine	41 (5)	76 (5)	97 (2)	102 (6)	106 (2)
O-DM-tramadol	39 (5)	67 (5)	89 (4)	96 (6)	97 (2)
Ethylmorphine	42 (5)	76 (5)	97 (4)	102 (2)	99 (1)
Tramadol	46 (5)	78 (3)	96 (3)	99 (1)	97 (1)
Pethidine	47 (5)	79 (4)	96 (4)	97 (1)	96 (2)
Ketobemidone	43 (5)	78 (7)	99 (4)	101 (3)	100 (2)
Buprenorphine	36 (6)	65 (7)	79 (5)	86 (5)	90 (3)
Fentanyl	38 (10)	66 (7)	79 (14)	83 (6)	93 (5)
Cyclopropylfentanyl	35 (11)	59 (7)	71 (20)	79 (9)	89 (6)
Etonitazepyne	24 (8)	38 (5)	49 (29)	54 (7)	68 (7)
Methadone	42 (8)	70 (5)	80 (18)	88 (3)	98 (5)

4.2 Extraction current

The extraction current was monitored to evaluate integrity of the liquid membrane and system stability. **Fig. S1** shows the recorded current in extraction of OF samples using a 1:2 (v/v) mixture of 6MC:Thy (1:2, molar ratio) and NPOE as the liquid membrane, applying 0 – 80 V for 20 min. The system was regarded as stable if the current was ≤ 50 μA per EME cell, and did not increase over time.

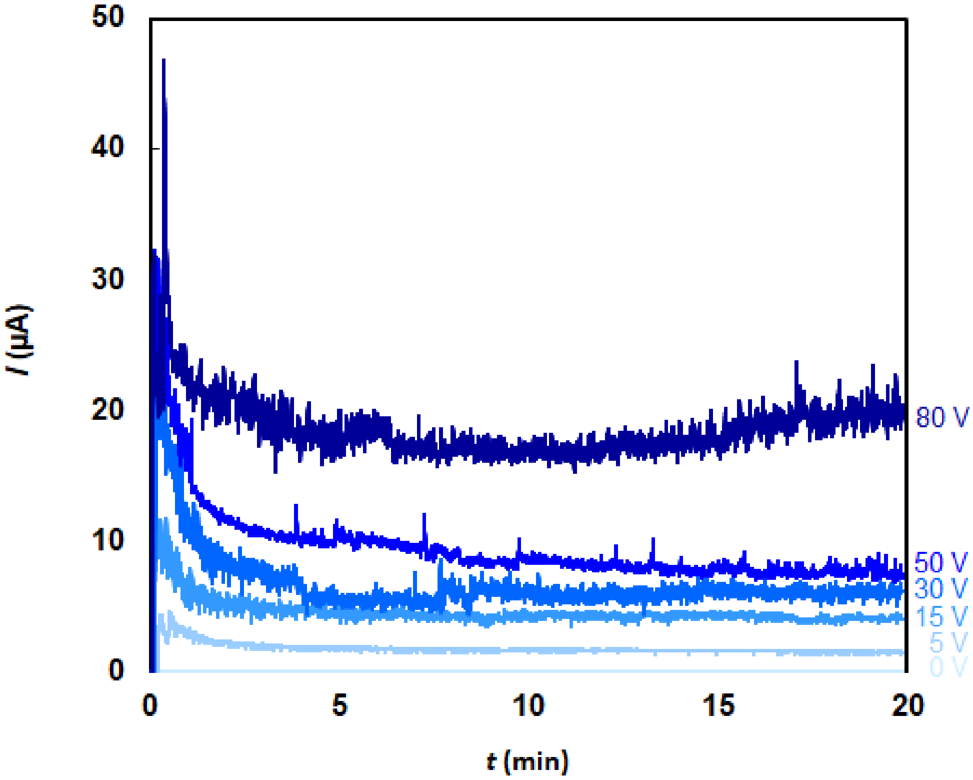

Figure S1: Current (*I*) per EME cell in extraction of OF samples applying 0 – 80 V for 20 min.
